# Supplementary material for: Smooth Interpolating Curves with Local Control and Monotone Alternating Curvature
Source: Comput Graph Forum. 2022 Oct 6;41(5):25–38. doi: 10.1111/cgf.14600 (PMC9827861; doi:10.1111/cgf.14600)
Supplement: Supplementary file 1 — Supplement Material [file CGF-41-25-s001.zip › Local-Smooth-Interpolating-MonoCurvature/extern/clothoids/docs/api-cpp/program_listing_file_Line.cc.html]

Program Listing for File Line.cc — Clothoids v2.0.9

### Navigation

- index
- toc
- Clothoids »
- Program Listing for File Line.cc

# Program Listing for File Line.cc¶

↰ Return to documentation for file (`Line.cc`)

```
/*--------------------------------------------------------------------------*\
 |                                                                          |
 |  Copyright (C) 2017                                                      |
 |                                                                          |
 |         , __                 , __                                        |
 |        /|/  \               /|/  \                                       |
 |         | __/ _   ,_         | __/ _   ,_                                |
 |         |   \|/  /  |  |   | |   \|/  /  |  |   |                        |
 |         |(__/|__/   |_/ \_/|/|(__/|__/   |_/ \_/|/                       |
 |                           /|                   /|                        |
 |                           \|                   \|                        |
 |                                                                          |
 |      Enrico Bertolazzi                                                   |
 |      Dipartimento di Ingegneria Industriale                              |
 |      Universita` degli Studi di Trento                                   |
 |      email: enrico.bertolazzi@unitn.it                                   |
 |                                                                          |
\*--------------------------------------------------------------------------*/

#include "Clothoids.hh"

// Microsoft visual studio Workaround
#ifdef max
  #undef max
#endif

#ifdef min
  #undef min
#endif

#include <algorithm>

namespace G2lib {

  using std::max;
  using std::min;
  using std::swap;

  LineSegment::LineSegment( BaseCurve const & C )
  : BaseCurve(G2LIB_LINE)
  {
    switch ( C.type() ) {
    case G2LIB_LINE:
      *this = *static_cast<LineSegment const *>(&C);
      break;
    case G2LIB_CIRCLE:
    case G2LIB_CLOTHOID:
    case G2LIB_BIARC:
    case G2LIB_BIARC_LIST:
    case G2LIB_CLOTHOID_LIST:
    case G2LIB_POLYLINE:
      UTILS_ERROR(
        "LineSegment constructor cannot convert from: {}\n",
        CurveType_name[C.type()]
      );
    }
  }

  #ifndef DOXYGEN_SHOULD_SKIP_THIS

  // - - - - - - - - - - - - - - - - - - - - - - - - - - - - - - - - - - - - - -
  typedef struct {
    real_type p[2];
    real_type q[2];
    real_type c;
    real_type s;
    real_type L;
  } L_struct;

  // - - - - - - - - - - - - - - - - - - - - - - - - - - - - - - - - - - - - - -
  // Given three colinear points p, q, r, the function checks if
  // point q lies on line segment 'pr'
  static
  bool
  onSegment(
    real_type const p[2],
    real_type const q[2],
    real_type const r[2],
    real_type const epsi
  ) {

    real_type mi_x, ma_x;
    if ( p[0] > r[0] ) { mi_x = r[0]; ma_x = p[0]; }
    else               { mi_x = p[0]; ma_x = r[0]; }

    bool ok = q[0] <= ma_x+epsi && q[0] >= mi_x-epsi;
    if ( ok ) {
      real_type mi_y, ma_y;
      if ( p[1] > r[1] ) { mi_y = r[1]; ma_y = p[1]; }
      else               { mi_y = p[1]; ma_y = r[1]; }
      ok = q[1] <= ma_y+epsi && q[1] >= mi_y-epsi;
    }
    return ok;
  }

  // To find orientation of ordered triplet (p, q, r).
  // The function returns following values
  // 0 --> p, q and r are collinear
  // 1 --> Clockwise
  // 2 --> Counterclockwise
  static
  int_type
  orientation(
    real_type const p[2],
    real_type const q[2],
    real_type const r[2],
    real_type const epsi
  ) {
    // See https://www.geeksforgeeks.org/orientation-3-ordered-points/
    // for details of below formula.
    real_type qp_x = q[0] - p[0];
    real_type qp_y = q[1] - p[1];
    real_type rq_x = r[0] - q[0];
    real_type rq_y = r[1] - q[1];

    real_type det = qp_y * rq_x - qp_x * rq_y;
    if ( std::abs(det) < epsi ) return 0;  // collinear
    return (det > 0)? 1: 2; // clock or counterclock wise
  }

  // - - - - - - - - - - - - - - - - - - - - - - - - - - - - - - - - - - - - - -

  static
  bool
  intersect(
    real_type        epsi,
    L_struct const & L1,
    L_struct const & L2,
    real_type      & s1,
    real_type      & s2
  ) {

    // Find the four orientations needed for general and special cases
    int_type o1 = orientation( L1.p, L1.q, L2.p, epsi );
    int_type o2 = orientation( L1.p, L1.q, L2.q, epsi );
    int_type o3 = orientation( L2.p, L2.q, L1.p, epsi );
    int_type o4 = orientation( L2.p, L2.q, L1.q, epsi );

    // General case
    if ( o1 != o2 && o3 != o4 ) {
      real_type det = L1.c * L2.s - L1.s * L2.c;
      real_type px  = L2.p[0]-L1.p[0];
      real_type py  = L2.p[1]-L1.p[1];
      s1 = (px * L2.s - py * L2.c)/ det;
      s2 = (px * L1.s - py * L1.c)/ det;
      return true;
    }

    // Special Cases
    // p1, q1 and p2 are collinear and p2 lies on segment p1q1
    if ( o1 == 0 && onSegment( L1.p, L2.p, L1.q, epsi ) ) {
      s1 = hypot( L2.p[0]-L1.p[0], L2.p[1]-L1.p[1] );
      s2 = 0;
      return true;
    }

    // p1, q1 and q2 are collinear and q2 lies on segment p1q1
    if ( o2 == 0 && onSegment( L1.p, L2.q, L1.q, epsi ) ) {
      s1 = hypot( L2.q[0]-L1.p[0], L2.q[1]-L1.p[1] );
      s2 = L2.L;
      return true;
    }

    // p2, q2 and p1 are collinear and p1 lies on segment p2q2
    if ( o3 == 0 && onSegment( L2.p, L1.p, L2.q, epsi ) ) {
      s1 = 0;
      s2 = hypot( L1.p[0]-L2.p[0], L1.p[1]-L2.p[1] );
      return true;
    }

    // p2, q2 and q1 are collinear and q1 lies on segment p2q2
    if ( o4 == 0 && onSegment( L2.p, L1.q, L2.q, epsi ) ) {
      s1 = L1.L;
      s2 = hypot( L1.q[0]-L2.p[0], L1.q[1]-L2.p[1] );
      return true;
    }

    s1 = s2 = 0;
    return false; // Doesn't fall in any of the above cases
  }

  // - - - - - - - - - - - - - - - - - - - - - - - - - - - - - - - - - - - - - -

  static
  bool
  collision(
    real_type        epsi,
    L_struct const & L1,
    L_struct const & L2
  ) {

    // Find the four orientations needed for general and special cases
    int_type o1 = orientation( L1.p, L1.q, L2.p, epsi );
    int_type o2 = orientation( L1.p, L1.q, L2.q, epsi );
    int_type o3 = orientation( L2.p, L2.q, L1.p, epsi );
    int_type o4 = orientation( L2.p, L2.q, L1.q, epsi );

    // General case
    if ( o1 != o2 && o3 != o4 ) return true;

    // Special Cases
    // p1, q1 and p2 are collinear and p2 lies on segment p1q1
    if ( o1 == 0 && onSegment( L1.p, L2.p, L1.q, epsi ) ) return true;

    // p1, q1 and q2 are collinear and q2 lies on segment p1q1
    if ( o2 == 0 && onSegment( L1.p, L2.q, L1.q, epsi ) ) return true;

    // p2, q2 and p1 are collinear and p1 lies on segment p2q2
    if ( o3 == 0 && onSegment( L2.p, L1.p, L2.q, epsi ) ) return true;

    // p2, q2 and q1 are collinear and q1 lies on segment p2q2
    if ( o4 == 0 && onSegment( L2.p, L1.q, L2.q, epsi ) ) return true;

    return false; // Doesn't fall in any of the above cases
  }

  #endif

  /*\
   |   _     _
   |  | |   (_)_ __   ___
   |  | |   | | '_ \ / _ \
   |  | |___| | | | |  __/
   |  |_____|_|_| |_|\___|
  \*/

  void
  LineSegment::build_2P(
    real_type x0,
    real_type y0,
    real_type x1,
    real_type y1
  ) {
    real_type dx = x1-x0;
    real_type dy = y1-y0;
    m_L      = hypot( dx, dy );
    m_x0     = x0;
    m_y0     = y0;
    m_theta0 = atan2(dy, dx);
    if ( m_L > 0 ) {
      m_c0 = dx / m_L;
      m_s0 = dy / m_L;
    } else {
      m_c0 = m_s0 = 0;
    }
  }

  // - - - - - - - - - - - - - - - - - - - - - - - - - - - - - - - - - - - - - -

  void
  LineSegment::bbox(
    real_type & xmin,
    real_type & ymin,
    real_type & xmax,
    real_type & ymax
  ) const {
    xmin = m_x0; xmax = m_x0+m_L*m_c0;
    ymin = m_y0; ymax = m_y0+m_L*m_s0;
    if ( xmin > xmax ) swap( xmin, xmax );
    if ( ymin > ymax ) swap( ymin, ymax );
  }

  // - - - - - - - - - - - - - - - - - - - - - - - - - - - - - - - - - - - - - -

  void
  LineSegment::bbox_ISO(
    real_type   offs,
    real_type & xmin,
    real_type & ymin,
    real_type & xmax,
    real_type & ymax
  ) const {
    real_type dx = offs*nx_Begin_ISO();
    real_type dy = offs*ny_Begin_ISO();
    xmin = m_x0+dx; xmax = xEnd()+dx;
    ymin = m_y0+dy; ymax = yEnd()+dy;
    if ( xmin > xmax ) swap( xmin, xmax );
    if ( ymin > ymax ) swap( ymin, ymax );
  }

  // - - - - - - - - - - - - - - - - - - - - - - - - - - - - - - - - - - - - - -

  void
  LineSegment::rotate( real_type angle, real_type cx, real_type cy ) {
    real_type dx  = m_x0 - cx;
    real_type dy  = m_y0 - cy;
    real_type C   = cos(angle);
    real_type S   = sin(angle);
    real_type ndx = C*dx - S*dy;
    real_type ndy = C*dy + S*dx;
    m_x0      = cx + ndx;
    m_y0      = cy + ndy;
    m_theta0 += angle;
    m_c0      = cos(m_theta0);
    m_s0      = sin(m_theta0);
  }

  // - - - - - - - - - - - - - - - - - - - - - - - - - - - - - - - - - - - - - -

  void
  LineSegment::reverse() {
    m_x0     += m_c0 * m_L;
    m_y0     += m_s0 * m_L;
    m_c0      = -m_c0;
    m_s0      = -m_s0;
    m_theta0 += Utils::m_pi;
    if ( m_theta0 > Utils::m_pi ) m_theta0 -= Utils::m_2pi;
  }

  // - - - - - - - - - - - - - - - - - - - - - - - - - - - - - - - - - - - - - -

  void
  LineSegment::paramNURBS( int_type & n_knots, int_type & n_pnts ) const {
    n_pnts  = 2;
    n_knots = 4;
  }

  // - - - - - - - - - - - - - - - - - - - - - - - - - - - - - - - - - - - - - -

  void
  LineSegment::toNURBS( real_type * knots, real_type Poly[][3] ) const {
    knots[0] = knots[1] = 0;
    knots[2] = knots[3] = 1;
    Poly[0][0] = m_x0;
    Poly[0][1] = m_y0;
    Poly[0][2] = 1;
    Poly[1][0] = m_x0+m_L*m_c0;
    Poly[1][1] = m_y0+m_L*m_s0;
    Poly[1][2] = 1;
  }

  // - - - - - - - - - - - - - - - - - - - - - - - - - - - - - - - - - - - - - -

  void
  LineSegment::toBS( real_type * knots, real_type Poly[2][2] ) const {
    knots[0] = knots[1] = 0;
    knots[2] = knots[3] = 1;
    Poly[0][0] = m_x0;
    Poly[0][1] = m_y0;
    Poly[1][0] = m_x0+m_L*m_c0;
    Poly[1][1] = m_y0+m_L*m_s0;
  }

  // - - - - - - - - - - - - - - - - - - - - - - - - - - - - - - - - - - - - - -

  bool
  LineSegment::intersect(
    LineSegment const & S,
    real_type         & s1,
    real_type         & s2
  ) const {
    // The main function that returns true if line segment 'p1q1'
    // and 'p2q2' intersect.
    L_struct L1;
    L_struct L2;

    L1.p[0] = xBegin();
    L1.p[1] = yBegin();
    L1.q[0] = xEnd();
    L1.q[1] = yEnd();
    L1.c    = m_c0;
    L1.s    = m_s0;
    L1.L    = m_L;

    L2.p[0] = S.xBegin();
    L2.p[1] = S.yBegin();
    L2.q[0] = S.xEnd();
    L2.q[1] = S.yEnd();
    L2.c    = S.m_c0;
    L2.s    = S.m_s0;
    L2.L    = S.m_L;

    real_type const epsi = max(m_L,S.m_L)*machepsi100;
    return G2lib::intersect( epsi, L1, L2, s1, s2 );
  }

  // - - - - - - - - - - - - - - - - - - - - - - - - - - - - - - - - - - - - - -

  bool
  LineSegment::intersect_ISO(
    real_type           offs,
    LineSegment const & S,
    real_type           S_offs,
    real_type         & s1,
    real_type         & s2
  ) const {
    // The main function that returns true if line segment 'p1q1'
    // and 'p2q2' intersect.
    L_struct L1;
    L_struct L2;

    L1.p[0] = xBegin_ISO(offs);
    L1.p[1] = yBegin_ISO(offs);
    L1.q[0] = xEnd_ISO(offs);
    L1.q[1] = yEnd_ISO(offs);
    L1.c    = m_c0;
    L1.s    = m_s0;
    L1.L    = m_L;

    L2.p[0] = S.xBegin_ISO(S_offs);
    L2.p[1] = S.yBegin_ISO(S_offs);
    L2.q[0] = S.xEnd_ISO(S_offs);
    L2.q[1] = S.yEnd_ISO(S_offs);
    L2.c    = S.m_c0;
    L2.s    = S.m_s0;
    L2.L    = S.m_L;

    real_type const epsi = max(m_L,S.m_L)*machepsi100;

    return G2lib::intersect( epsi, L1, L2, s1, s2 );
  }

  // - - - - - - - - - - - - - - - - - - - - - - - - - - - - - - - - - - - - - -

  bool
  LineSegment::collision( LineSegment const & S ) const {
    // The main function that returns true if line segment 'p1q1'
    // and 'p2q2' intersect.
    L_struct L1;
    L_struct L2;

    L1.p[0] = xBegin();
    L1.p[1] = yBegin();
    L1.q[0] = xEnd();
    L1.q[1] = yEnd();
    L1.c    = m_c0;
    L1.s    = m_s0;
    L1.L    = m_L;

    L2.p[0] = S.xBegin();
    L2.p[1] = S.yBegin();
    L2.q[0] = S.xEnd();
    L2.q[1] = S.yEnd();
    L2.c    = S.m_c0;
    L2.s    = S.m_s0;
    L2.L    = S.m_L;

    real_type const epsi = max(m_L,S.m_L)*machepsi100;

    return G2lib::collision( epsi, L1, L2 );
  }

  // - - - - - - - - - - - - - - - - - - - - - - - - - - - - - - - - - - - - - -

  bool
  LineSegment::collision_ISO(
    real_type           offs,
    LineSegment const & S,
    real_type           S_offs
  ) const {
    // The main function that returns true if line segment 'p1q1'
    // and 'p2q2' intersect.
    L_struct L1;
    L_struct L2;

    L1.p[0] = xBegin_ISO(offs);
    L1.p[1] = yBegin_ISO(offs);
    L1.q[0] = xEnd_ISO(offs);
    L1.q[1] = yEnd_ISO(offs);
    L1.c    = m_c0;
    L1.s    = m_s0;
    L1.L    = m_L;

    L2.p[0] = S.xBegin_ISO(S_offs);
    L2.p[1] = S.yBegin_ISO(S_offs);
    L2.q[0] = S.xEnd_ISO(S_offs);
    L2.q[1] = S.yEnd_ISO(S_offs);
    L2.c    = S.m_c0;
    L2.s    = S.m_s0;
    L2.L    = S.m_L;

    real_type const epsi = max(m_L,S.m_L)*machepsi100;

    return G2lib::collision( epsi, L1, L2 );
  }

  /*\
   |   _       _                          _
   |  (_)_ __ | |_ ___ _ __ ___  ___  ___| |_
   |  | | '_ \| __/ _ \ '__/ __|/ _ \/ __| __|
   |  | | | | | ||  __/ |  \__ \  __/ (__| |_
   |  |_|_| |_|\__\___|_|  |___/\___|\___|\__|
  \*/

  /*\
   |        _                     _   ____       _       _
   |    ___| | ___  ___  ___  ___| |_|  _ \ ___ (_)_ __ | |_
   |   / __| |/ _ \/ __|/ _ \/ __| __| |_) / _ \| | '_ \| __|
   |  | (__| | (_) \__ \  __/\__ \ |_|  __/ (_) | | | | | |_
   |   \___|_|\___/|___/\___||___/\__|_|   \___/|_|_| |_|\__|
  \*/

  int_type
  LineSegment::closestPoint_ISO(
    real_type   qx,
    real_type   qy,
    real_type & x,
    real_type & y,
    real_type & s,
    real_type & t,
    real_type & dst
  ) const {

    real_type dx = qx - m_x0;
    real_type dy = qy - m_y0;
    s = dx * tx_Begin() + dy * ty_Begin();
    t = dx * nx_Begin_ISO() + dy * ny_Begin_ISO();

    if ( s < 0 ) { // distanza sul bordo 0
      s = 0;
      x = m_x0;
      y = m_y0;
    } else if ( s > m_L ) {
      s = m_L;
      eval( s, x, y );
    } else {
      dst = std::abs(t);
      eval( s, x, y );
      return 1;
    }

    dx  = qx-x;
    dy  = qy-y;
    t   = dx * nx_Begin_ISO() + dy * ny_Begin_ISO();
    dst = hypot( dx, dy );
    return -1;
  }

  // - - - - - - - - - - - - - - - - - - - - - - - - - - - - - - - - - - - - - -

  int_type
  LineSegment::closestPoint_ISO(
    real_type   qx,
    real_type   qy,
    real_type   offs,
    real_type & x,
    real_type & y,
    real_type & s,
    real_type & t,
    real_type & dst
  ) const {
    real_type xx0 = m_x0+offs*nx_Begin_ISO();
    real_type yy0 = m_y0+offs*ny_Begin_ISO();

    real_type dx = qx - xx0;
    real_type dy = qy - yy0;
    s = dx * tx_Begin() + dy * ty_Begin();
    t = dx * nx_Begin_ISO() + dy * ny_Begin_ISO();

    if ( s < 0 ) { // distanza sul bordo 0
      s = 0;
      x = xx0;
      y = yy0;
    } else if ( s > m_L ) {
      s = m_L;
      eval_ISO( s, offs, x, y );
    } else {
      t  += offs;
      dst = std::abs(t);
      eval_ISO( s, offs, x, y );
    }

    dx  = qx-x;
    dy  = qy-y;
    t   = dx * nx_Begin_ISO() + dy * ny_Begin_ISO() + offs;
    dst = hypot( dx, dy );
    return -1;
  }

  // - - - - - - - - - - - - - - - - - - - - - - - - - - - - - - - - - - - - - -

  ostream_type &
  operator << ( ostream_type & stream, LineSegment const & c ) {
    fmt::print( stream,
      "x0     = {}\n"
      "y0     = {}\n"
      "theta0 = {}\n"
      "L      = {}\n",
      c.m_x0, c.m_y0, c.m_theta0, c.m_L
    );
    return stream;
  }

  // - - - - - - - - - - - - - - - - - - - - - - - - - - - - - - - - - - - - - -

}

// EOF: Line.cc
```

### Quick search

### Table of Contents

- Matlab Interface Manual
- C++ API
- MATLAB API

«
hide menu

menu
sidebar
»

### Navigation

- index
- toc
- Clothoids »
- Program Listing for File Line.cc

© Copyright 2021, Enrico Bertolazzi and Marco Frego.
Created using Sphinx 4.2.0.
